# Supplementary material for: Predicting visual function by interpreting a neuronal wiring diagram
Source: Nature. 2024 Oct 2;634(8032):113–23. doi: 10.1038/s41586-024-07953-5 (PMC11446822; doi:10.1038/s41586-024-07953-5)
Supplement: Supplementary file 6 — CRF and ERF predictions for individual TmY4 and TmY9 cells. Analogous to Supplementary Data 3, but for TmY target types. Shown are the top four monosynaptic pathways, the strongest pathway passing through each of the top ten intermediary types (ranking from Extended Data Fig. 7), and the trisynaptic pathway Tm1–TmY–Dm3–TmY (see the section entitled Prediction of spatial normalization). [file 41586_2024_7953_MOESM6_ESM.zip › DataS4/TmY9q/720575940624353277.pdf]

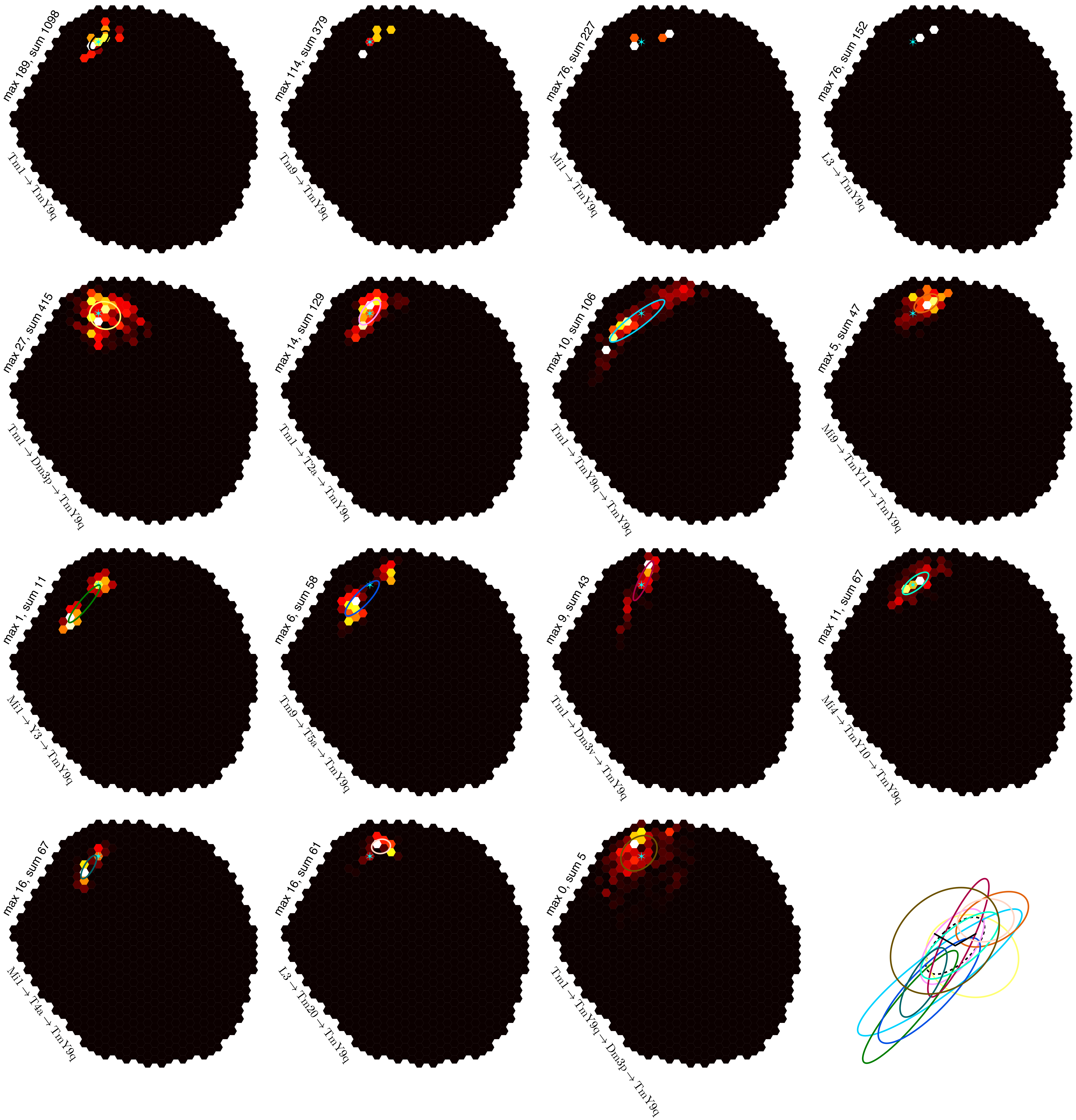
max 189, sum 1098
Tm1 → TmY9q
max 114, sum 379
Tm9 → TmY9q
max 76, sum 227
Mi1 → TmY9q
max 76, sum 152
L3 → TmY9q
max 27, sum 415
Tm1 → Dm3p → TmY9q
max 14, sum 129
Tm1 → T2a → TmY9q
max 10, sum 106
Tm1 → TmY9q → TmY9q
max 5, sum 47
Mi9 → TmY11 → TmY9q
max 1, sum 11
Mi1 → Y3 → TmY9q
max 6, sum 58
Tm9 → T5a → TmY9q
max 9, sum 43
Tm1 → Dm3v → TmY9q
max 11, sum 67
Mi4 → TmY10 → TmY9q
max 16, sum 67
Mi1 → T4a → TmY9q
max 16, sum 61
L3 → Tm20 → TmY9q
max 0, sum 5
Tm1 → TmY9q → Dm3p → TmY9q
